# Life History and the Relation Between Population Dynamics and Meteorological Factors of *Hyphantria cunea* (Lepidoptera: Erebidae: Arctiidae) in Shanghai, China

**DOI:** 10.3390/insects16111136

**Published:** 2025-11-06

**Authors:** Siqi Tang, Zichun Li, Guangyu Huang, Yangyang Han, Dejun Hao

**Affiliations:** 1 Co-Innovation Center for the Sustainable Forestry in Southern China, College of Forestry and Grassland, Nanjing Forestry University, Nanjing 210037, China; siqitarn@njfu.edu.cn (S.T.); leezichun@njfu.edu.cn (Z.L.); 2 Jinshan Forestry Work Station of Shanghai, Shanghai 201599, China; zyang0young@163.com; 3 Shanghai Forestry Station, Shanghai 200072, China; 15380905949@163.com

**Keywords:** invasive species, insect development, meteorological factors, correlation analysis, seasonal dynamics, Shanghai

## Abstract

The fall webworm *Hyphantria cunea* (Lepidoptera: Erebidae) is a globally invasive species that causes serious damage to agriculture and forestry. Since the first invasion reported in Liaoning Province, China, it has spread extensively southward and was found in Shanghai in 2019. Owing to the climatic and environmental differences between these regions, the biology and population dynamics of *H. cunea* may have undergone changes in its newly invaded areas. In this study, we investigated the biology of *H. cunea* in Shanghai and monitored the population dynamics using sex-pheromone-baited traps. We analyzed the relationships between population dynamics and local meteorological factors, including temperature, precipitation, and sunshine duration. Our result indicates high temperatures and large diurnal temperature ranges have significant effects (*R*^2^ = 0.723, *p* < 0.001) on the survival and development of *H. cunea*. Consequently, they play an important role in shaping its population dynamics. These findings enhance our understanding of how *H. cunea* responds to Shanghai’s environmental conditions, provide a scientific basis for local pest management, and contribute to predicting its population trends.

## Simple Summary

The fall webworm *Hyphantria cunea* is an invasive insect that causes significant damage to crops and trees. The invasion of this species in China began in the Northeast and spread southward. It was recently reported in Shanghai in southeast China. Due to the differences in climate and environment, the insect’s biology and population dynamics may have changed in Shanghai. In this study, we investigated the life cycle and population dynamics of the fall webworm in Shanghai and monitored adult numbers using pheromone-baited traps. We also analyzed how local weather affects its growth, survival, and reproduction. Our results indicate that both high temperatures and daily temperature fluctuations strongly influence survival and development, shaping population dynamics throughout the year. These findings improve our understanding of how the fall webworm responds to environmental conditions in Shanghai, provide a scientific basis for local management strategies, and offer insights for predicting its potential spread.

## 1. Introduction

The fall webworm, *H. cunea* (Lepidoptera: Erebidae), native to North America, is a globally invasive insect pest that causes extensive damage to both agriculture and forestry. Its rapid expansion across Europe and Asia has been attributed to strong ecological adaptability, high reproductive potential, flight capacity, and human activities such as transoceanic trade and transportation [1,2]. When invasive species colonize a new region, changes in environmental factors can lead to substantial alterations in their morphology, behavior, and life-history traits [3]. Morphological variation in invasive organisms is well documented, such as the appearance of head spines in invasive Daphnia in North America when predator pressure is high [4]. Similarly, invasive species may exhibit behavioral shifts in their new ranges; for example, virile crayfish shifted towards more aggressive behavior in their invasive range compared to their native range [5].

In China, *H. cunea* was first introduced into Liaoning Province in the Northeast and subsequently spread southward and westward [6]. In 2019, it was reported for the first time in Shanghai. Given the considerable latitudinal difference, the climatic conditions in Shanghai differ greatly from the initial invasion area. Northern China is characterized by a temperate continental climate with lower temperatures and less precipitation, while southern China, including Shanghai, has a subtropical monsoon climate with higher temperatures and humidity. Moreover, in Shanghai, *H. cunea* has been frequently recorded feeding on *Taxodium distichum* and *Metasequoia glyptostroboides*, both of which contain abundant terpenoid secondary metabolites [7]. These factors suggest that the biology and population dynamics of *H. cunea* may have undergone substantial changes in this newly invaded region. To improve the management strategies for this pest in Shanghai, it is therefore necessary to clarify its biological traits and population dynamics under local conditions.

The growth and development of *H. cunea* are influenced by meteorological factors such as temperature, precipitation, and photoperiod, which in turn shape its population dynamics [8,9,10,11]. Temperature plays a crucial role in the development of *H. cunea*. The lower developmental threshold and thermal constant for one generation are 10.6 °C and 724.4 degree days, respectively [12]. Within the range of 21–27 °C, rising temperatures shorten larval duration while increasing larval weight and female fecundity [13]. However, both excessively high and low temperatures have detrimental effects on its development [14,15]. Relative humidity is another key factor. Low humidity decreases egg survival rate and molting [16,17], whereas excessive moisture causes pupation failure [18]. Photoperiod further regulates development, particularly diapause, which is induced under short-day conditions, with night length being crucial [11]. In addition, larval foraging occurs primarily at night [9]. Collectively, these meteorological factors affect the development and survival of *H. cunea*, thereby shaping its population dynamics, though studies in Shanghai remain limited.

To improve the management strategies for *H. cunea* in Shanghai, we investigated the biology of different developmental stages of *H. cunea* populations in Shanghai. Using sex-pheromone-baited traps, we monitored population dynamics and analyzed the relationships of the population with local meteorological variables, including daily maximum and minimum temperatures, 10-day average temperature, 10-day average precipitation, monthly number of rainy days, and 10-day average sunshine duration. Our results characterized the biological traits of *H. cunea* in Shanghai and revealed that high temperatures and precipitation significantly affect its population dynamics. These findings provide a scientific basis for the effective management of *H. cunea* in Shanghai and offer a reference for predicting its population dynamics in other regions.

## 2. Materials and Methods

### 2.1. Investigation on the Biology of H. cunea in Shanghai

To investigate the larval biology, *H. cunea* egg masses were collected from the cypress forest stands (*T. distichum* and *M. glyptostroboides*) at three locations in Shanghai, including Luxiang Town, Jinshan District (121.18° E, 30.84° N), Maogang Town, Songjiang District (121.14° E, 30.95° N), and Liantang Town, Qingpu District (121.08° E, 30.99° N). The experiments were conducted as a semi-field experiment. The eggs were placed in outdoor rearing cages (40 cm × 40 cm × 40 cm, metal frames covered with fine mesh), which allowed them to experience natural environmental conditions, and freshly collected *T. distichum* leaves were provided as food. Developmental duration, larval body length, and head capsule width were recorded daily.

To investigate the biological characteristics of pupal and adults, the pupae of *H. cunea* were collected from the same three monitoring sites: Luxiang Town, Jinshan District (121.18° E, 30.84° N); Maogang Town, Songjiang District (121.14° E, 30.95° N); and Liantang Town, Qingpu District (121.08° E, 30.99° N). Their body sizes were measured, and each individual was then placed outdoors in a 35 mm Petri dish, allowing exposure to natural environmental factors. Emergence was observed at 30 min intervals. The sex, eclosion time, emergence rate, and parasitism rate were recorded. For emerged adults, body length and wing span were measured.

### 2.2. Monitoring of H. cunea Population Dynamics in Shanghai

A total of 88 monitoring sites were established across Minhang (121.38° E, 31.12° N), Jiading (121.27° E, 31.38° N), Baoshan (121.48° E, 31.41° N), Pudong (121.56° E, 31.22° N), Jinshan (121.35° E, 31.74° N), Songjiang (121.23° E, 31.03° N), Qingpu (121.13° E, 31.15° N), and Fengxian (121.47° E, 31.92° N) districts in Shanghai, including 29 three-year monitoring sites and 59 two-year monitoring sites (Table S1). At each site, 30 Unitrap-type traps were installed. Traps were spaced at least 500 m apart from each other and hung at a height of no less than 2 m. Each trap was baited with a rubber septum loaded with synthetic *H. cunea* sex pheromones (purchased from Nanjing Zhilin Biotechnology Co., Ltd., Nanjing, China). Lures were replaced every three months [19]. The number of captured male moths was recorded every five days, and the traps were cleaned by removing captured moths, debris, and leaves.

### 2.3. Meteorological Data

Meteorological data were obtained from the China Meteorological Administration and meteorological stations in each district of Shanghai. The dataset included seven meteorological factors recorded from 2021 to 2023: 10-day average temperature (°C), 10-day average maximum temperature (°C), 10-day average minimum temperature (°C), 10-day average temperature range (°C), number of rainy days per 10 days, 10-day total precipitation (mm), and 10-day total sunshine duration (h).

### 2.4. Statistical Analysis

Differences in eclosion rhythms of *H. cunea* adults were analyzed using Duncan’s new multiple range test. For convenience in data analysis, temperatures were converted from °C to Kelvin (K), although all temperatures are reported in °C in the manuscript. To assess the interactive effects among the seven meteorological factors: 10-day average temperature (X_1_), 10-day average maximum temperature (X_2_), 10-day average minimum temperature (X_3_), 10-day average diurnal temperature range (X_4_), number of rainy days per 10 days (X_5_), 10-day total precipitation (X_6_), and 10-day total sunshine duration (X_7_), a principal component analysis (PCA) was performed based on the correlation matrix. The total variance explained, component matrix, loading matrix, and score matrix were subsequently obtained. Considering that the larval stage of *H. cunea* lasts approximately 30 days, the relationships between meteorological factors and the average trap catch 30 days later were examined using linear regression analysis, followed by ANOVA to test the significance of the regression model. In view of the voltinism of *H. cunea*, only data within the 30-day period surrounding each population peak were included in the analysis. All analyses were carried out using SPSS 26.0 (IBM SPSS Statistics, Chicago, IL, USA). Graphs and charts were plotted using ORIGINPRO 2022b (OriginLab Inc., Northampton, UK).

## 3. Result

### 3.1. Biology of H. cunea in Shanghai

The morphological characteristics and occurrence periods of *H. cunea* populations in Shanghai were recorded, including larval body length, head capsule width, adult body length, wingspan, and the initial appearance of each larval generation (Table S2).

In Shanghai, the first-instar larvae of the first generation appeared from 10–15 May. Those of the second generation appeared from 1–6 July. The first-instar larvae of the third (overwintering) generation appeared from 20–25 August. The first generation of *H. cunea* larvae had six instars, with the mature larvae exhibiting head capsule widths of 2.3–2.5 mm (2.37 ± 0.04 mm) and body lengths of 26.0–27.0 mm (26.58 ± 0.17 mm). The second generation had seven instars, and the mature larvae had head capsule widths ranging from 2.3 to 2.5 mm (2.39 ± 0.04 mm) and body lengths ranging from 22.5 to 26.0 mm (24.38 ± 0.67 mm). The third (overwintering) generation also had seven instars. In the mature larvae of the third generation, the head capsule width ranged from 2.4 to 2.8 mm (2.57 ± 0.13 mm), and the body length ranged from 28.0 to 33.0 mm (30.50 ± 0.79 mm).

**Figure 1:**
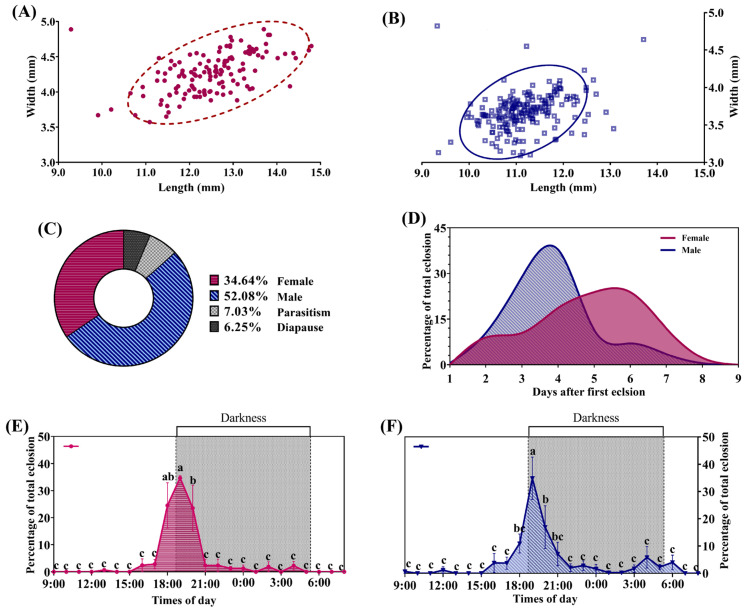
Morphological characteristics of pupae and eclosion rhythm of *H. cunea* in Shanghai: (**A**) Female pupae length and width. (**B**) Male pupae length and width. (**C**) Proportions of *H. cunea* pupae by sex, diapause, and parasitism rate. (**D**) Temporal trend of adult eclosion of *H*. *cunea*, showing the proportion of adults emerging each day relative to total emergence. (**E**) Eclosion rhythm of female adults. (**F**) Eclosion rhythm of male adults. (**E**,**F**) were analyzed using Duncan’s new multiple range test. Statistically significant differences are indicated by different lowercase letters (*p* < 0.05).

The pupae of *H. cunea* females measured approximately 10.5–15.3 mm in length (12.53 ± 0.89 mm) and 3.8–5.0 mm in width (4.26 ± 0.28 mm) (Figure 1A). Male pupae were 10.0–13.0 mm long (11.20 ± 0.28 mm) and 3.0–4.0 mm wide (3.67 ± 0.24 mm) (Figure 1B). Overall, females were larger than males in body size. The natural parasitism rate of pupae was 6.25%, and the female-to-male sex ratio was 0.665 (Figure 1C).

The overwintering generation of *H. cunea* adults exhibited larger body sizes. Females measured 14.5–16.9 mm (15.78 ± 0.52 mm) in body length and 36.5–43.8 mm (40.37 ± 1.70 mm) in wingspan. Males measured 13.1–15.8 mm (14.56 ± 0.62 mm) in body length and 29.2–33.3 mm (31.89 ± 0.89 mm) in wingspan (Table S2). In contrast, the first-generation adults were smaller, with females 11.4–13.5 mm (12.39 ± 0.45 mm) long and wingspans of 30.8–37.0 mm (34.20 ± 1.49 mm), and males 10.2–12.9 mm (11.51 ± 0.63 mm) long and wingspans of 23.4–29.9 mm (26.92 ± 1.53 mm) (Table S2).

Male emergence peaked on the third and fourth days after the first emergence was observed, accounting for about 40% of the total eclosed males, whereas female emergence peaked on the fifth or sixth days (Figure 1D). Female emergence occurred mainly around sunset (19:00), peaking within one hour before and after it. Male emergence showed two peaks: a smaller one around sunrise (5:00) and a major one near sunset (19:00) (Figure 1E,F).

### 3.2. Population Dynamics of H. cunea in Shanghai

**Figure 2:**
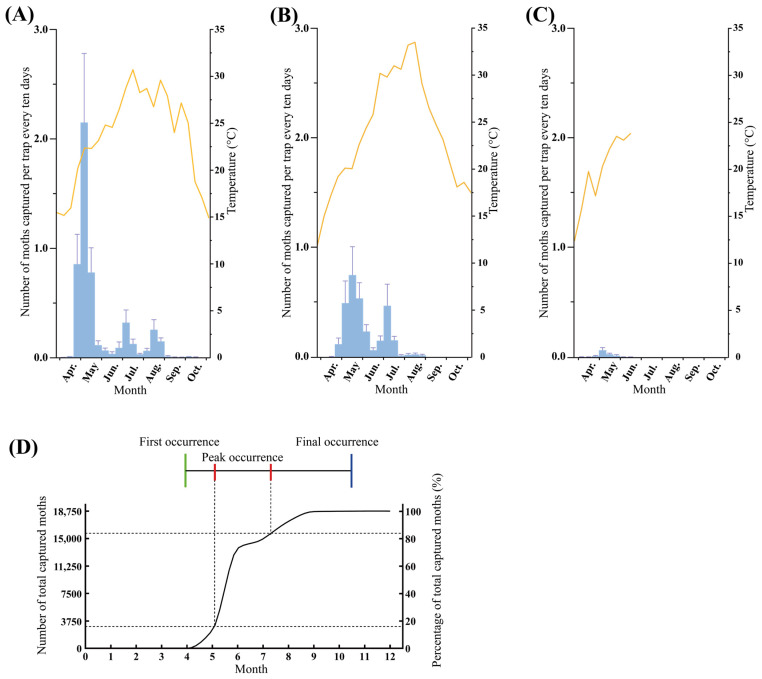
Population dynamics of *H. cunea* in Shanghai: (**A**) Population dynamics of *H. cunea* and temperature in Shanghai in 2021, (**B**) Population dynamics of *H. cunea* and temperature in 2022, (**C**) Population dynamics of *H. cunea* and temperature in 2023, (**D**) Occurrence pattern of *H. cunea* adults in Shanghai. In (**A**–**C**), the left *Y*-axis and blue bars represent the number of moths captured, while the right *Y*-axis and orange line represent the 10-day average temperature. Moths captured data in (**A**–**C**) are presented as means + SE.

In Shanghai, the first appearance of *H. cunea* adults occurred consistently in early to mid-April across three consecutive years (7 April 2021, 12 April 2022, and 6 April 2023). In 2021 and 2022, three distinct population peaks were observed, corresponding to the overwintering, first, and second generations (Figure 2A,B). The overwintering generation peaked from late April to early May, the first generation from late June to early July, and the second generation from early to late August, with the last adults observed in mid-October (Figure 2). However, only the overwintering generation was clearly distinguishable in 2023 (Figure 2C). The population density in 2021 was exceptionally high, particularly for the overwintering generation, while the first and second generations were relatively smaller (Figure 2A). Notably, third-generation adults were possibly recorded in October 2021 with an average of 0.0076 moths per trap. In 2022, the overwintering and first generations had comparable population levels, whereas the second generation had much lower population (Figure 2B). In 2023, only the adults of the overwintering generation were captured, with extremely low abundance—average trap catches were below 0.1 moths per trap throughout the year (Figure 2C). Overall, the annual peak occurrence of *H. cunea* in Shanghai extended from early May to mid-July, and the last captures were recorded in mid to late October (Figure 2D).

### 3.3. Principal Component Analysis of Meteorological Factors

**Figure 3:**
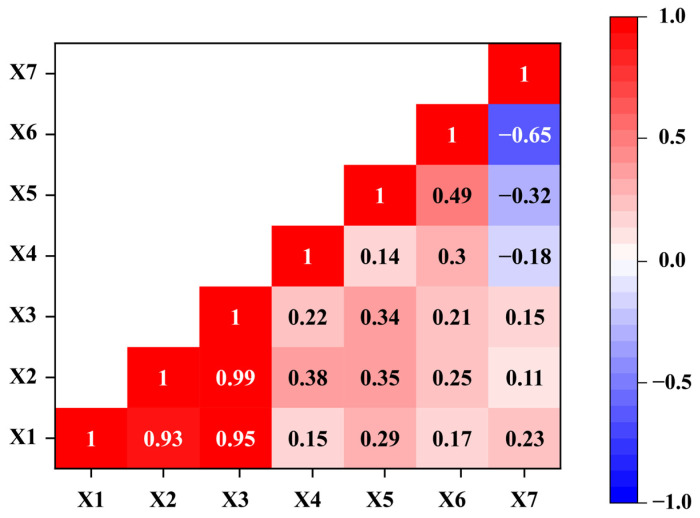
Correlation of different meteorological factors. Both the horizontal and vertical axes represent seven meteorological variables (X1–X7). The colors indicate the correlation coefficients between the variables: Red represents a positive correlation, Blue represents a negative correlation and White or light colors indicate a weak correlation.

Correlation analysis indicated that the 10-day average temperature (X_1_), 10-day average maximum temperature (X_2_), and 10-day average minimum temperature (X_3_) were strongly correlated, while the number of rainy days per 10 days (X_5_), 10-day total precipitation (X_6_), and 10-day total sunshine duration (X_7_) also showed moderate correlations (Figure 3). In contrast, the 10-day average diurnal temperature range (X_4_) appeared relatively independent (Figure 3).

According to Table 1, the first (F_1_) and second (F_2_) principal components together explained 74.96% of the total variance. Although the eigenvalue of the third component (F_3_) was below 1, it was retained to increase the cumulative explained variance to 87.74%.

According to the rotated factor matrix (Table 2), the 10-day average temperature (X_1_), 10-day average maximum temperature (X_2_), and 10-day average minimum temperature (X_3_) showed the highest loadings on F_1_. Since these three variables represent temperature indicators, F_1_ was designated as Temperature Level. The number of rainy days per 10 days (X_5_), 10-day total precipitation (X_6_), and 10-day total sunshine duration (X_7_) had the highest loadings on F_2_; therefore, F_2_ was named as Precipitation. The 10-day average diurnal temperature range (X_4_) exhibited the highest loading on F_3_, and F_3_ was accordingly designated as Diurnal Temperature Range.

Based on the factor score coefficient matrix, the expressions for the principal components were derived as follows:

F1 = 0.330X1 + 0.298X2 + 0.324X3 − 0.053X4 + 0.118X5 − 0.010X6 + 0.147X7(1)

F2 = -0.035X1 − 0.026X2 − 0.010X3 − 0.097X4 + 0.405X5 + 0.424X6 − 0.435X7(2)

F3 = -0.094X1 + 0.117X2 − 0.045X3 + 0.949X4 − 0.343X5 + 0.057X6 − 0.059X7(3)

The weights for constructing a comprehensive meteorological index were determined based on each principal component’s proportion of the cumulative variance.

F = 0.504F1 + 0.324F2 + 0.173F3(4)

This comprehensive index (F) can be used to represent the overall meteorological condition.

### 3.4. Correlation Between Meteorological Factors and Population Dynamics of H. cunea in Shanghai

**Figure 4:**
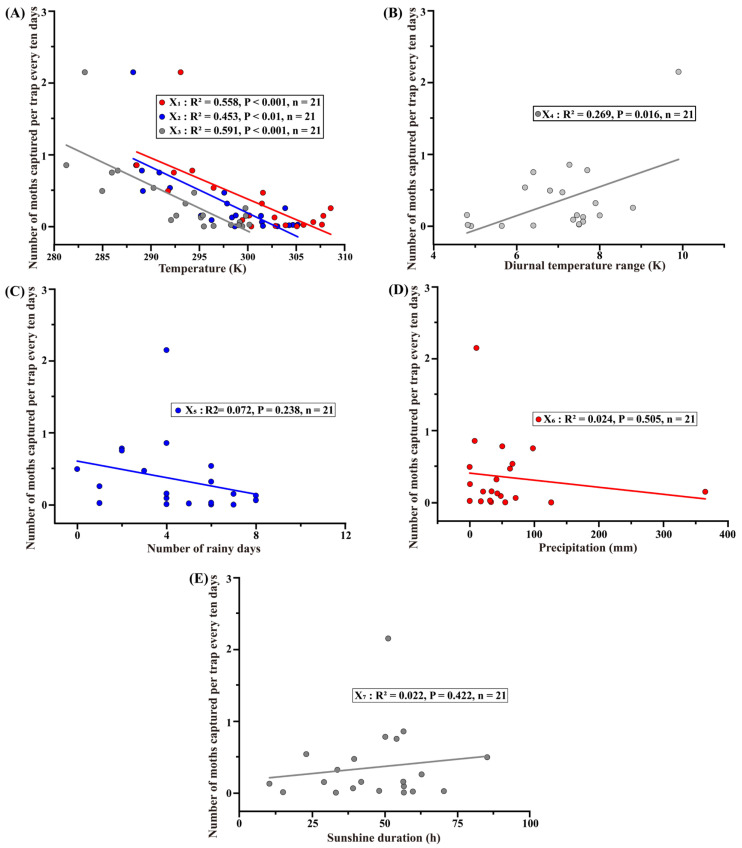
Linear regression analyses of the relationships between meteorological factors and the population dynamics of *H. cunea* in Shanghai: (**A**) Relationship between temperature and population dynamics of *H. cunea*. (**B**) Relationship between diurnal temperature range and population dynamics of *H. cunea*. (**C**) Relationship between the number of rainy days and the population dynamics of *H. cunea*. (**D**) Relationship between precipitation and population dynamics of *H. cunea*. (**E**) Relationship between sunshine duration and population dynamics of *H. cunea.* In (**A**), X_1_ represents the 10-day average temperature, X_2_ the 10-day average maximum temperature, and X_3_ the 10-day average minimum temperature. In (**B**), X_4_ represents the 10-day average diurnal temperature range. In (**C**), X_5_ represents the number of rainy days per 10 days. In (**D**), X_6_ represents the 10-day total precipitation, and in (**E**), X_7_ represents the 10-day total sunshine duration. The solid lines in different colors represent the fitted linear regression relationships between the number of moths captured and the corresponding environmental variables.

The 10-day average temperature (Figure 4A, *R*^2^ = 0.558, *p* < 0.001, *n* = 21), 10-day average maximum temperature (Figure 4A, *R*^2^ = 0.453, *p* < 0.001, *n* = 21), and 10-day average minimum temperature (Figure 4A, *R*^2^ = 0.591, *p* < 0.01, *n* = 21) were all significantly negatively correlated with the average number of *H. cunea* adults trapped 30 days later, indicating that higher temperatures may substantially reduce adult abundance. In contrast, the 10-day average diurnal temperature range (Figure 4B, *R*^2^ = 0.453, *p* < 0.01, *n* = 21) showed a significant positive correlation with the average trap catch 30 days later, suggesting that larger temperature fluctuations favor the development or survival of *H. cunea*. Meanwhile, the number of rainy days per 10 days (Figure 4C, *R*^2^ = 0.024, *p* = 0.505, *n* = 21), 10-day total precipitation (Figure 4D, *R*^2^ = 0.072, *p* = 0.238, *n* = 21), and 10-day total sunshine duration (Figure 4E, *R*^2^ = 0.022, *p* = 0.524, *n* = 21) showed no significant correlation with the average number of *H. cunea* adults trapped 30 days later. While the regressions revealed clear relationships between temperature variables and adult abundance, the small sample size (*n* = 21) may limit the reliability of effect size estimates and their applicability to other populations or years.

Based on the results of PCA and linear regression analysis, 10-day average minimum temperature (X_3_) and 10-day average diurnal temperature range (X_4_) were selected as predictors for further fitting. The results showed that the model including both X_3_ and X_4_ exhibited better performance (*R*^2^ = 0.723) and had a significant effect on *H. cunea* population dynamics (ANOVA, *F*(2,18) = 23.547, *p* < 0.001). Moreover, no multicollinearity (VIF = 1.048) was detected between the two factors (Table 3).

## 4. Discussion

Compared to the higher animals, such as the vertebrates, insects usually have short generation cycles and high reproductive rates, making them highly sensitive to environmental changes [20]. Such environmental fluctuations are often accompanied by phenotypic variation [21]. When invasive species colonize a new region, changes in environmental factors can lead to substantial alterations in their morphology, behavior, and life-history traits [3]. Therefore, understanding the biology and behavioral patterns of invasive species in newly colonized regions is essential for developing effective management strategies. In this study, we investigated the biology and population dynamics of *H. cunea* in its newly invaded region of Shanghai, China. We also analyzed the relationships between the population dynamics of *H. cunea* and local meteorological factors. The results indicated that temperature and diurnal temperature range were the primary factors influencing the population dynamics of *H. cunea*. These findings provide a theoretical basis for predicting its occurrence and developing effective control strategies.

The overall morphology of *H. cunea* adults observed in our experiment was consistent with previous reports [22]. Notably, adults of the overwintering generation were larger than those of the first and second generations (Table S2). This difference is likely due to the greater energy reserves required for overwintering; these individuals tend to accumulate more fat, carbohydrates, and other nutrients, resulting in larger body size [23,24]. In addition, most adult emergences occurred around dusk (Figure 1E,F). This is consistent with previous observations that emergence peaks approximately one hour after sunset and may be triggered by the decreasing light intensity [25].

We found that the overwintering, first, and second generations of adult *H. cunea* in Shanghai could be clearly distinguished in 2021 and 2022 (Figure 2A,B). However, adult moths were still captured in October 2021, suggesting the potential presence of third-generation adults and indicating that up to four generations of adults may occur per year (Figure 2B). The voltinism of *H. cunea* is highly variable, ranging from one to five generations in its native range. Across China, it varies from one to four generations from south to north, likely influenced by photoperiod and temperature [1,26]. Shanghai, situated at a relatively low latitude, experiences higher temperatures than northern regions. Insect growth and development are strongly temperature-dependent, with growth rates generally increasing as ambient temperature rises [27,28,29]. Shanghai experiences relatively warmer conditions. These conditions may promote the development of *H. cunea* and potentially lead to a greater number of generations per year [30].

The trap catch numbers suggested that the population of *H. cunea* in Shanghai gradually declined from 2021 to 2023. Within each year, successive generations also tended to decrease in population size (Figure 2A–C). This trend may be attributed to the increasingly frequent extreme high-temperature events associated with progressive climate warming. The population sizes of the first and second generations in 2021 were comparable (Figure 2A), while in 2022, the second generation showed a marked decline relative to the first (Figure 2B). This decline was likely caused by an extreme heat wave. From late July to mid-August in Shanghai, the average high temperature reached 37 °C, with peaks up to 40 °C. These temperatures were substantially higher than those recorded in 2021. This extreme heat wave may have contributed to the decline in *H. cunea* populations. This effect is supported by previous studies showing that prolonged heat stress can greatly reduce their survival rate [31].

Linear regression analysis revealed a strong negative correlation between population dynamics and temperature. This indicates that excessively high temperatures may adversely affect the development and occurrence of *H. cunea* (Figure 4A). At present, the southward expansion of *H. cunea* in China is largely restricted to the middle and lower reaches of the Yangtze River. This may be due to the high temperatures in this region during midsummer, from mid-July to mid-August, which coincide with the late larval and pupation stages of *H. cunea*. During this period, thermal stress can reduce reproductive capacity [31], while low humidity can impede physiological processes such as pupation and molting [16], ultimately limiting further spread. However, continued vigilance is needed against the southward spread of *H. cunea*. The species exhibits strong adaptability and is widely distributed in its native range, from Canada to Mexico [1]. Moreover, it can be unintentionally transported through human activities such as the timber trade and material movement. Such human-assisted dispersal could allow the species to leapfrog into regions further south of its current distribution [32].

Temperature fluctuations can have profound effects on insect development, survival, and reproduction. In the present study, regression analysis revealed a positive correlation between the population dynamics of *H. cunea* and the diurnal temperature range (Figure 4B). Previous studies have also demonstrated the physiological effects of temperature fluctuations on insects [33,34,35]. For example, under mean temperatures of 25 °C and 27 °C with diurnal variations of ±3 °C and ±5 °C, the reproductive performance of the Mediterranean fruit fly (*Ceratitis capitata*) was significantly higher than that observed under constant temperatures [36]. Studies on metabolism and cellular structure have shown that, compared with constant temperatures, appropriate temperature fluctuations can enhance insect survival. Moreover, these fluctuations can also increase the development rate and thermal tolerance. These beneficial effects are possibly mediated by the up-regulation of key proteins involved in glycolysis, the tricarboxylic acid cycle, ATP synthesis, and molecular transport [37]. Furthermore, diel temperature cycles act as important zeitgebers that influence insect circadian rhythms. These rhythms, in turn, play critical roles in regulating various physiological and behavioral activities [38,39,40].

To better assess the influence of meteorological factors on the population dynamics of *H. cunea*, a multiple regression analysis was conducted. The analysis included variables that showed significant effects on trap catch numbers, such as the 10-day average temperature, 10-day average maximum temperature, 10-day average minimum temperature, and 10-day average diurnal temperature range. However, strong correlations were found among the 10-day average temperature, 10-day average maximum temperature, and 10-day average minimum temperature (Figure 3). To reduce model complexity and multicollinearity, only the 10-day average maximum temperature, which exhibited the best model performance, was retained as one of the predictors. The 10-day average diurnal temperature range was included as the second variable in the multiple linear regression model. The resulting regression model demonstrated a good fit, with an *R*^2^ value of 0.723, explaining approximately 72.3% of the variance in the dependent variable (Table 2). This indicates that the model effectively captures the climatic conditions influencing the occurrence of *H. cunea*. It can therefore be used to make preliminary predictions of its population dynamics based on meteorological data. However, because *H. cunea* is multivoltine and environmental factors are highly variable, some influences, such as humidity, air pressure, and wind, remain unaccounted for. These limitations highlight the need for further investigation [41].

## 5. Conclusions

This study clarified the biology and population dynamics of *H. cunea* in Shanghai, providing valuable guidance for its management in the region. Correlation analyses between meteorological factors and population fluctuations revealed that temperature and diurnal temperature range significantly affect *H. cunea* abundance. A multiple linear regression model was established to predict the occurrence of *H. cunea* based on these climatic factors. However, some factors influencing population size remain unidentified, and further research is needed to better understand and control the spread of this species.

## Figures and Tables

**Table 1:** Total variance explained.

Initial Eigenvalues				Rotation Sums of Squared Loadings			
Factor	Eigenvalues	Percentage of Variance (%)	Cumulative (%)	Factor	Eigenvalues	Percentage of Variance (%)	Cumulative (%)
F_1_	3.294	47.060	47.060	F_1_	3.095	44.208	44.208
F_2_	1.953	27.900	74.960	F_2_	1.988	28.395	72.602
F_3_	0.895	12.780	87.740	F_3_	1.060	15.138	87.740
F_4_	0.532	7.603	95.343				
F_5_	0.270	3.861	99.205				
F_6_	0.056	0.795	100.000				
F_7_	3.423 × 10^−8^	4.889 × 10^−7^	100.000				

**Table 2:** Factor loadings, rotated factor loadings, and factor score coefficient matrix of the seven meteorological factors.

	Factor Matrix			Rotated Factor Matrix			Factor Score Coefficient Matrix		
Factor	F1	F2	F3	F1	F2	F3	F1	F2	F3
X_1_	0.910	0.330	−0.106	0.974	0.008	0.015	0.330	−0.035	−0.094
X_2_	0.969	0.196	0.074	0.960	0.095	0.229	0.298	−0.026	0.117
X_3_	0.949	0.259	−0.071	0.981	0.074	0.073	0.324	−0.010	−0.045
X_4_	0.401	−0.296	0.836	0.167	0.147	0.948	−0.053	−0.097	0.949
X_5_	0.528	−0.480	−0.409	0.371	0.715	−0.165	0.118	0.405	−0.343
X_6_	0.434	−0.784	−0.061	0.136	0.860	0.217	−0.010	0.424	0.057
X_7_	−0.028	0.898	0.047	0.283	−0.837	−0.170	0.147	−0.435	−0.059

10-day average temperature (X_1_), 10-day average maximum temperature (X_2_), 10-day average minimum temperature (X_3_), 10-day average diurnal temperature range (X_4_), number of rainy days per 10 days (X_5_), 10-day total precipitation (X_6_), and 10-day total sunshine duration (X_7_).

**Table 3:** Results of regression analysis between *H. cunea* population dynamics and meteorological factors.

Dependent Variable		Meteorological Factors		Regression Equation	Coefficient of Determination	ANOVA	VIF
Y	The numbers of moths captured per trap every 10 days	X_3_	10-day average minimum temperature	Y = −0.057X_3_ − 0.144X_4_ + 16.154	*R*^2^ = 0.723	*F* = 23.547 *p* < 0.001	1.048
		X_4_	10-day average diurnal temperature range				

## Data Availability

The original contributions presented in this study are included in the article/Supplementary Materials. Further inquiries can be directed to the corresponding author.

## Supplementary Materials

The following supporting information can be downloaded at: https://www.mdpi.com/article/10.3390/insects16111136/s1, Table S1: Trap deployment of Hyphantria cunea in Shanghai, 2021–2023; Table S2: Morphological characteristics and developmental periods of Hyphantria cunea; Table S3: Meteorological Data; Table S4: Monitoring Records of Hyphantria cunea.
