# Supplementary material for: Life History and the Relation Between Population Dynamics and Meteorological Factors of Hyphantria cunea (Lepidoptera: Erebidae: Arctiidae) in Shanghai, China
Source: Insects. 2025 Nov 6;16(11):1136. doi: 10.3390/insects16111136 (PMC12653576; doi:10.3390/insects16111136)
Supplement: Supplementary file 1 [file insects-16-01136-s001.zip › Table S1.pdf]

Table S1. Trap deployment of *Hyphantria cunea* in Shanghai, 2021–2023.

Year	Administrative district	Number of monitoring sites	Number of traps	Tree age (years)	Canopy density
2021	Baoshan district	4	120	5-20	0.5-0.6
	Jiading district	9	270	15-30	0.7-0.8
	Pudong district	24	720	5-10	0.3-0.45
	Jinshan district	10	300	5-30	0.3-0.8
	Songjiang district	15	450	5-25	0.3-0.7
	Qingpu district	7	210	5-25	0.3-0.7
	Fengxian district**+**	8	240	5-30	0.4-0.8
	Minhang district**+**	11	330	5-25	0.4-0.7
2022	Minhang district	6	180	5-25	0.4-0.7
	Jinshan district	9	270	15-30	0.7-0.8
	Pudong district	10	300	5-10	0.3-0.45
	Jinshan district	11	330	5-30	0.3-0.8
	Songjiang district	10	300	5-25	0.3-0.7
	Qingpu district	8	240	5-25	0.3-0.7
	Fengxian district	5	150	5-30	0.4-0.8
2023	Minhang district	3	150	5-25	0.4-0.7
	Baoshan district	1	30	5-20	0.5-0.6
	Jiading district	7	210	15-30	0.7-0.8
	Pudong district	5	150	5-10	0.3-0.45
	Jinshan district	5	150	5-30	0.3-0.8
	Songjiang district	6	180	5-25	0.3-0.7
	Qingpu district	5	150	5-25	0.3-0.7
	Fengxian district	1	30	5-30	0.4-0.8

+ indicates the newly infected areas of the *H. cunea* in the current year.
