# Supplementary material for: Life History and the Relation Between Population Dynamics and Meteorological Factors of Hyphantria cunea (Lepidoptera: Erebidae: Arctiidae) in Shanghai, China
Source: Insects. 2025 Nov 6;16(11):1136. doi: 10.3390/insects16111136 (PMC12653576; doi:10.3390/insects16111136)
Supplement: Supplementary file 1 [file insects-16-01136-s001.zip › Table S2.pdf]

Table S2. Morphological characteristics and developmental periods of *Hyphantria cunea.*

**Generation**	**Sex**	**Instars**	**Body length (mm)**	**Head capsule width (mm)**	**Wingspan (mm)**	Occurrence period
First generation larvae	/	1	1.8 ~ 2.5	0.23 ~ 0.33	/	10 ~ 15 May
	/	2	4.0 ~ 4.4	0.40 ~ 0.46	/	16 ~ 21 May
	/	3	7.9 ~ 8.9	0.80 ~ 0.85	/	24 ~ 27 May
	/	4	13.5 ~ 15	1 ~ 1.4	/	28 ~ 31 May
	/	5	19.5 ~ 22.2	1.7 ~ 2	/	1 ~ 4 June
	/	6	26 ~ 27	2.3 ~ 2.5	/	4 ~ 12 June
First generation adults	Female	/	11.4 ~ 13.5	/	30.8 ~ 37.0	21 ~ 25 June
	Male	/	10.2 ~ 12.9	/	23.4 ~ 29.9	20 ~ 27 June
Second generation larvae	/	1	0.8 ~ 1.2	0.17 ~ 0.24	/	1 ~ 6 July
	/	2	4.1 ~ 5.1	0.36 ~ 0.44	/	5 ~ 10 July
	/	3	5.5 ~ 9.0	0.6 ~ 1.1	/	11 ~ 14 July
	/	4	12.05 ~ 14.5	1.5 ~ 1.8	/	14 ~ 19 July
	/	5	16.7 ~ 19.5	2.0 ~ 2.1	/	19 ~ 20 July
	/	6	19.1 ~ 22.0	2.0 ~ 2.2	/	21 ~ 27 July
	/	7	22.5 ~ 26.0	2.3 ~ 2.5	/	27 July ~ 8 August
Second generation adults	Female	/	13.9 ~ 14.9	/	34.5 ~ 38.8	12 August ~ 4 September
	Male	/	11.5 ~ 13.8	/	26.2 ~ 31.3	10 ~ 30 August
Third generation Larvae (overwintering)	/	1	2.2 ~ 3.0	0.17 ~ 0.24	/	20 ~ 25 August
	/	2	3.6 ~ 4.3	0.30 ~ 0.41	/	23 ~ 27 August
	/	3	7.2 ~ 8.5	0.60 ~ 0.81	/	25 ~ 30 August
	/	4	10.0 ~ 13.5	0.95 ~ 1.1	/	28 August ~ 1 September
	/	5	19.2 ~ 21.0	1.7 ~ 2.0	/	1 ~ 3 September
	/	6	22.2 ~ 24.0	1.8 ~ 2.1	/	3 ~ 7 September
	/	7	28.0 ~ 33.0	2.4 ~ 2.8	/	6 ~ 13 September
overwintering adults	Female	/	14.5 ~ 16.9	/	36.5 ~ 43.8	10 ~ 20 May (the following year)
	Male	/	13.1 ~ 15.8	/	29.2 ~ 33.3	6 ~ 12 May (the following year)
